# Intracranial Ewing Sarcoma in Pregnancy: Anesthetic and Clinical Challenges

**DOI:** 10.7759/cureus.88677

**Published:** 2025-07-24

**Authors:** Syeda Mariam Zehra Naqvi, Mehwish Shafique, Asma Akram, Huma Saleem

**Affiliations:** 1 Anesthesiology, Shaukat Khanum Memorial Cancer Hospital and Research Centre, Lahore, Lahore, PAK

**Keywords:** ewing sarcoma, intracranial tumor, neuroanesthesia, obstetric anesthesia, pregnancy

## Abstract

We report an unusual case of a 26-year-old female patient diagnosed with primary intracranial Ewing sarcoma (ES) at 16 weeks' gestation. She had vertigo, vomiting, and worsening visual disturbances. MRI showed a solid-cystic mass in the left occipital area with a midline shift. Neurosurgical resection was performed under general anesthesia, with careful intraoperative management to ensure maternal stability and fetal safety. Unique considerations in our anesthesia technique for pregnancy were the management of cerebral blood flow, increased intracranial pressure, and avoiding teratogenic medications. Throughout the intraoperative period, maternal hemodynamic parameters were closely tailored, at times requiring transfusion due to significant bleeding. Postoperative CT imaging showed standard imaging changes consistent with surgical resection and no immediate complications. Fetal monitoring via obstetric ultrasound was normal. Results of histopathology confirmed ES. Here, we discuss the rarity of an intracranial ES occurring during pregnancy and the contours of anesthesia practice in this patient with a complex perioperative process. Coordinating care and developing an individualized plan is crucial to support the best outcome for the mother and fetus.

## Introduction

Intracranial Ewing sarcoma (ES) represents an exceptionally uncommon and highly aggressive tumor. While ES is more commonly found in bones, it can occasionally emerge in soft tissues or internal organs. Although cases involving the brain are exceedingly rare, a few reports in the literature have described its presence in atypical and unexpected anatomical regions [1].

ES is most frequently observed in children and young adults and is characterized by rapid progression, a high recurrence rate, and substantial metastatic potential. Primary intracranial ES is postulated to originate from mesenchymal cells of the dura or leptomeninges [2]. Its occurrence during pregnancy is exceptionally uncommon, presenting unique challenges due to the dual need for maternal and fetal safety [3].

The management of intracranial ES during pregnancy requires a delicate balance between effective cancer treatment and minimizing fetal risks. Headache, visual changes, and focal neurological signs could all be related to pregnancy and may lead to a delayed diagnosis [3]. Combination therapy involving surgery, chemotherapy, and radiation is conventional; however, pregnancy complicates the treatment plan because both maternal and fetal health should be appropriately balanced. The rarity of primary intracranial sarcomas, including ES, restricts the availability of comprehensive data and standardized treatment guidelines, particularly when these tumors occur during pregnancy [4].

This case report illustrates a rare instance of primary intracranial ES diagnosed during pregnancy. It highlights the diagnostic and therapeutic dilemmas posed and raises awareness about the anesthetic techniques employed during surgery to maintain both maternal and fetal health.

## Case presentation

A 26-year-old female, weighing 66 kg with a BMI of 27, presented at 16 weeks of gestation (four months pregnant) with complaints of vertigo, vomiting, and progressive eyesight weakness. She reported experiencing these symptoms for the past month. On examination, she exhibited neck pain, bilateral abducens palsy, and papilledema.

**Figure 1:**
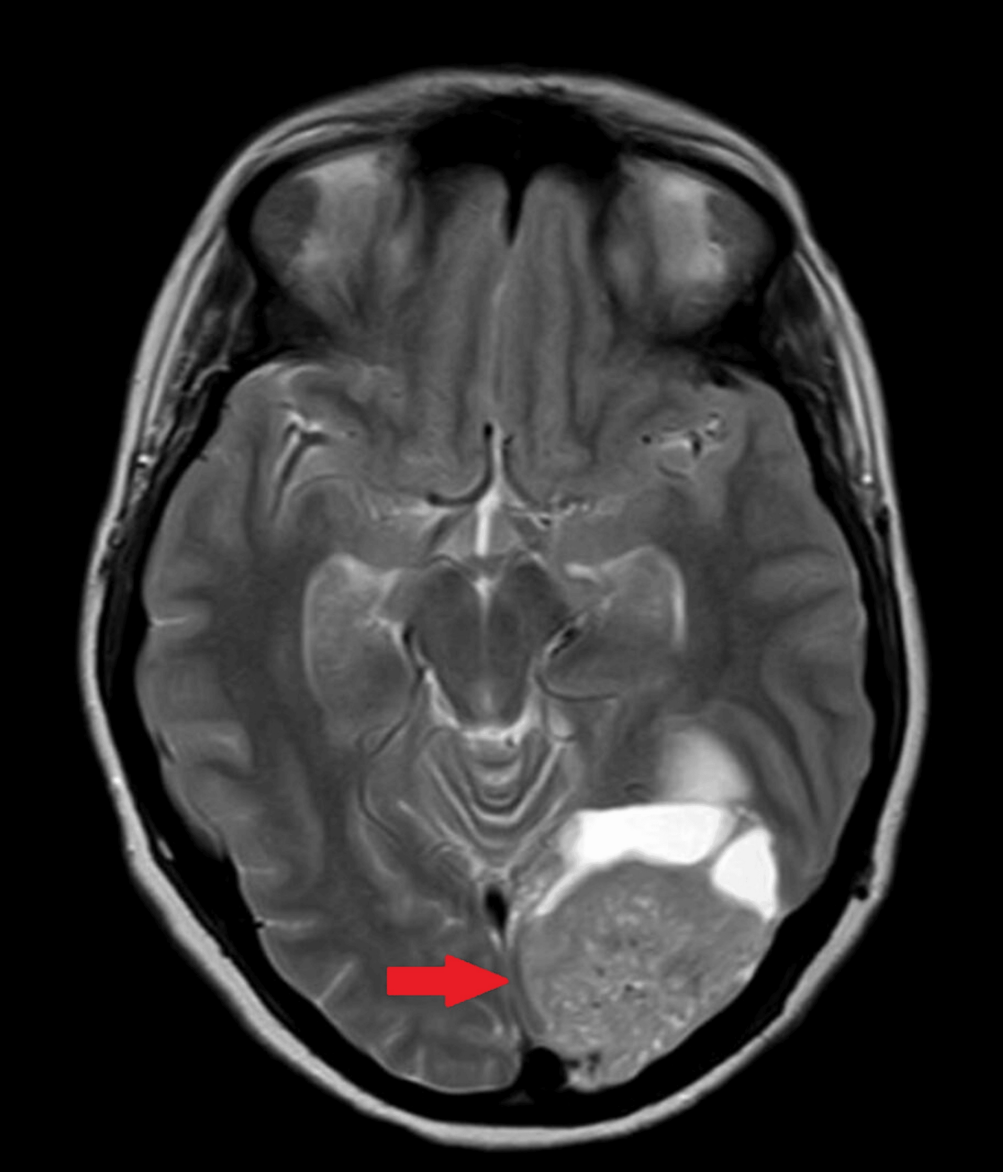
Preoperative axial T2-weighted MRI brain scan showing left occipital solid-cystic mass with midline shift and inferior extension (red arrow).

An MRI without contrast performed revealed a solid-cystic mass in the left occipital region with a 7 mm midline shift towards the right and sprouting at the foramen magnum (Figure 1).

The initial differential diagnosis included pleomorphic xanthoastrocytoma or pilocytic astrocytoma. On repeat scan, a diagnosis of low-grade glioma was established. As per the multidisciplinary team (MDT) consensus, surgical intervention was planned. It was decided to proceed with neuronavigation-guided left occipital craniotomy and maximum safe resection of the lesion.

During the pre-operative assessment, the patient was noted to have no comorbidities, with a Glasgow Coma Scale (GCS) score of 15/15. No new significant findings were noted on systemic clinical examination at that time. Laboratory investigations and ECG results were within normal limits. A pre-operative gynecological evaluation by the obstetrics team determined that no special positioning was required during surgery as the gestational age was less than 20 weeks. They also recommended keeping oxytocin and misoprostol available during and after surgery to address any potential obstetric complications.

On arrival at the operating room, the patient was pre-medicated with 100 mcg fentanyl, and anesthesia induction was performed using fentanyl, propofol, and atracurium. The airway was secured with a reinforced endotracheal tube, and two wide-bore intravenous (IV) cannulas were placed. A left radial artery arterial line was secured for continuous monitoring. For analgesia, a scalp block was performed using 20 mL of 0.5% bupivacaine. Maintenance was done with the inhalational agent sevoflurane. We avoided medications with known teratogenic risks, including benzodiazepines and nonsteroidal anti-inflammatory drugs (NSAIDs). Following smooth induction of anesthesia, the patient was positioned prone with adequate care. Chest and pelvic supports were placed, and the abdomen was left free to ensure optimal positioning and reduce pressure on the fetus. Her head was stabilized in a Mayfield clamp (Integra LifeSciences, Princeton, NJ, USA).

Intraoperatively, analgesia was maintained with paracetamol and dexmedetomidine infusion at a dose of 0.2 to 1 µg/kg/hour. Blood sugar levels were checked twice. Phenylephrine infusion was required to manage blood pressure; it was continued for four hours and tapered at the end of the procedure. Arterial blood gas (ABG) analysis revealed a drop in hematocrit from 31% preoperatively to 23% intraoperatively. Given the drop in hematocrit and the requirement for phenylephrine infusion, two packed red blood cell (PRBC) units, totaling 600 mL, were transfused. The total intraoperative duration was approximately seven hours. The tumor findings were extremely vascular, with hemorrhage into the tumor-associated cyst. The total resection of the lesion was successfully achieved. Intraoperative frozen section analysis was not performed.

At the conclusion of the surgery, the patient was repositioned supine, and extubation was performed once she was awake and responsive. She was then transferred to the post-anesthesia care unit (PACU) with stable vitals. Post-transfusion hemoglobin levels were ordered and reported to be 11 g/dL, matching the preoperative value. Blood sugar levels and an obstetric ultrasound (OB scan) were also performed. The OB scan showed a single live intrauterine fetus with good cardiac activity.

**Figure 2:**
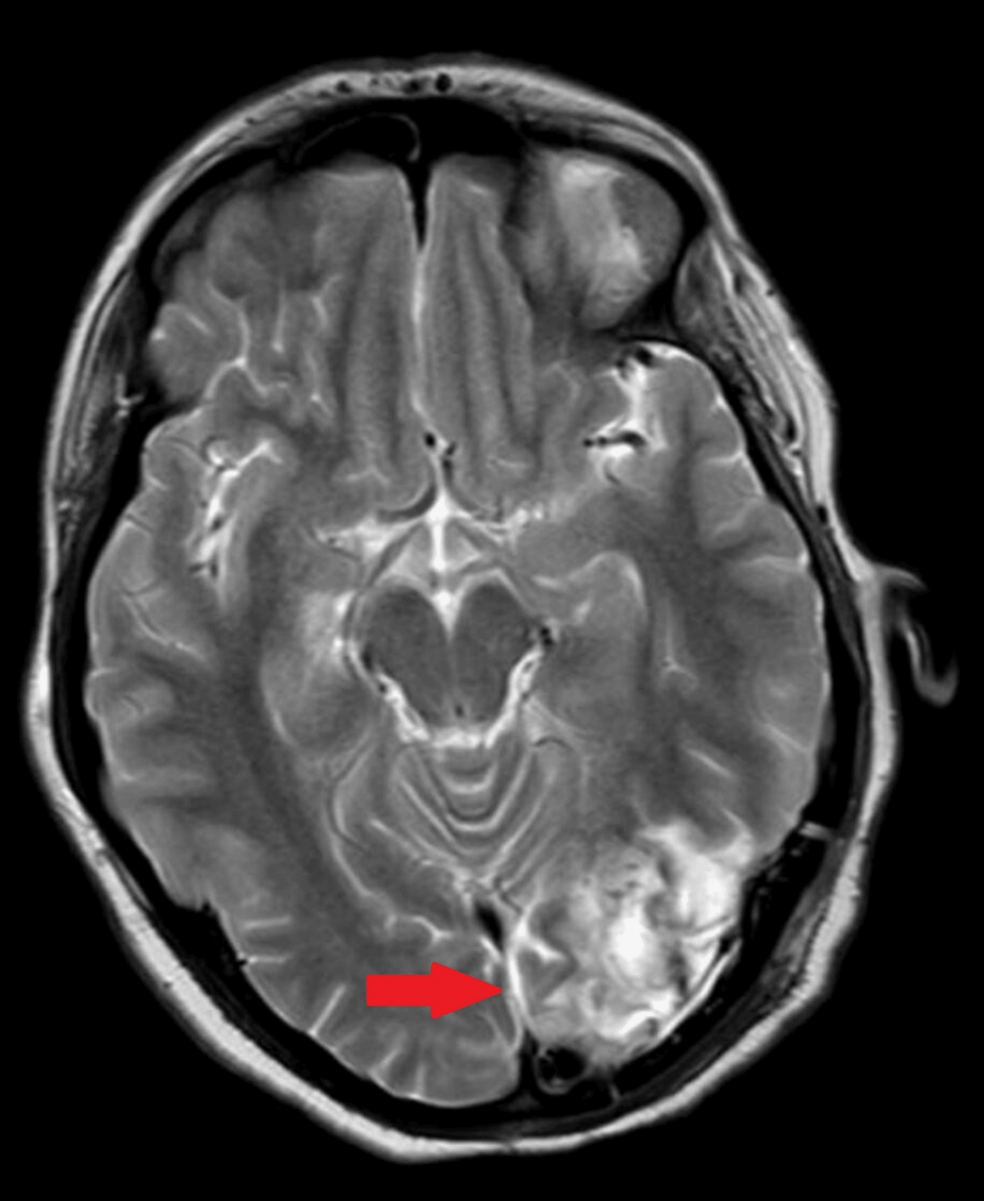
Postoperative axial T2-weighted MRI brain scan showing parenchymal changes in the left occipital region consistent with post-surgical effects (red arrow).

Post-craniotomy MRI revealed postoperative changes in the left occipital region with underlying brain parenchymal heterogeneity and the possibility of subacute infarction (Figure 2).

In the ICU, a targeted bedside OB scan was performed, confirming fetal well-being. An MDT meeting was conducted following the biopsy report, which revealed a malignant neoplasm composed of small, round cells arranged in sheets. The tumor cells exhibited scant cytoplasm and round nuclei, confirming the diagnosis of ES. The team recommended a whole-body MRI to assess metastasis; however, due to the patient’s pregnancy, this was not feasible. Assistance was sought from the radiology department, which proposed an alternative plan of an MRI brain and whole spine without contrast. Additionally, MRI scans of both upper and lower limbs without contrast and CT thorax (abdominal cover) were recommended to localize potential metastatic lesions while ensuring maternal and fetal safety.

MRI upper and lower limbs revealed no evidence of metastasis. No osseous or soft tissue lesions or suspicious abnormal masses were detected.

Following the neurosurgery team's recommendations, the patient was referred to the oncology and gynecology-obstetrics clinic. The oncologist recommended testing for the EWSR1 gene mutation, and a PET-CT scan was advised. In the event that the patient chose to terminate the pregnancy, a treatment plan involving 14 cycles of adjuvant chemotherapy was recommended, using the VDC/IE regimen (vincristine, doxorubicin, and cyclophosphamide alternating with ifosfamide and etoposide). The potential side effects of chemotherapy, including fertility concerns, were discussed with the family.

The patient remains under close monitoring, with ongoing MDT evaluations to determine the next course of action for her treatment, balancing maternal and fetal well-being.

## Discussion

Non-obstetric surgery is seen in 2% of pregnant women, 42% during the first trimester, and 35% during the second trimester. Anesthesia during pregnancy involves physiological changes such as modified drug effects, increased blood volume, and decreased oxygen stores. These challenges involve the management of hypotension, provision of oxygenation, prevention of teratogenic drugs, and reducing fetal risk, all of which are essential to maternal and fetal security [5].

Maternal cardiac output increases by 50% by mid-pregnancy due to an increase in heart rate and stroke volume, with reduced vascular resistance [6]. Oxygen demand increases 60% at term, which is accompanied by a 45% increase in minute ventilation. Progesterone-induced hyperventilation leads to mild respiratory alkalosis to prevent fetal acidosis. Pregnancy decreases functional residual capacity (FRC), which predisposes to rapid desaturation during intubation. Airway management is also difficult because of more soft tissue and vocal cord edema. Pre-oxygenation is very important and is done preferably in a slightly head-up position, keeping in mind that there are chances of failed intubation. Intrauterine asphyxia is a significant danger during maternal surgery because it leads to maternal hypoxia, hypercarbia, hypocarbia, hypotension, and causes an increase in uterine tone. Maternal oxygenation and hemodynamic stability are very important because they can prevent fetal acidosis and hypoxia. End-tidal CO_2_ measurement and ABG analysis should be done because both hypercarbia and hypocarbia can be harmful to the fetus [7].

Neuroanesthesia in pregnancy requires management of maternal changes and fetal protection. The major issues are adequate cerebral perfusion to prevent hypotension and intracranial pressure (ICP). Accurate fluid management and vasopressor therapy must be initiated to maintain the cerebral perfusion pressure (CPP) and the ICP in equilibrium. Situations that can lead to elevated ICP, such as intubation or neck manipulation, can be avoided, particularly in subsequent stages of pregnancy when uterine pressure has already started interfering with venous return and cerebral perfusion. Transient mild hyperventilation can be used to decrease ICP as needed. Agents must be titrated, and opioids must be avoided whenever possible. Supine positioning is avoided, and continuous fetal monitoring must be maintained [8].

Prone positioning during neuroanesthesia in pregnancy is difficult due to the growing uterus, which occludes the inferior vena cava and lowers venous return, leading to hypotension and impaired fetal and maternal perfusion. Even minimal lateral tilt or wedges can stop this. Ventilation needs to be maintained at normocapnia to avoid such issues as uteroplacental vasoconstriction. The prone position also increases ICP risk and exacerbates airway management, as intubation and neck movement will be harder. In addition, facial and ocular pressure could compromise ocular perfusion. Continuous fetal monitoring and proper padding to avoid pressure injuries are mandatory [9].

Anesthetic management of intracranial ES patients has unique challenges related to tumor site, metastasis, and side effects of treatment. Preoperative neurological evaluation is important, as the tumor can lead to headache, seizures, or increased ICP. The management of raised ICP included head elevation, hyperventilation, and osmotic diuretics. Tumor bulk or previous surgery may make airway management difficult, and preparation for difficult intubation is required. Cerebral perfusion intraoperatively should be maintained, with careful blood pressure and ICP monitoring and management through normocapnic ventilation and head elevation. Anesthetic agents, like sevoflurane or propofol, should be titrated to avoid hypotension, and opioids should be used cautiously due to potential respiratory depression. Surgical considerations, like blood loss, need to be managed carefully. Postoperatively, continual neurological monitoring should be done in order to detect complications such as seizures or bleeding. Pain management should be done through multimodal strategies, avoidance of excessive use of opioids, and respiratory support may be demanded of patients with a compromise at baseline. Infection control is most important, particularly with immunocompromised hosts, with prophylactic antibiotics and vigilant monitoring for sepsis.

## Conclusions

The anesthetic management of resection of intracranial ES is complicated by a number of considerations, including elevated ICP, airway concerns, and the need to ensure cerebral perfusion. Preoperative evaluation, ICP monitoring, and postoperative management are vital to prevent neurological injury and to maintain adequate hemodynamics. Opioid administration should be matched to alternatives to allow the management of pain without respiratory depression. Cooperation of neurosurgeons, oncologists, and intensivists is a formula for success. In parturients, anesthetic management additionally includes considerations for maternal physiologic changes, fetal status, and deliberate drug selection to help ensure cerebral perfusion and limit prospective maternal and fetal risk.
